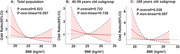# Age‐dependent relationship between body mass index and cognitive impairment: A cross‐sectional study based on the rural population aged 40 years old and above in Xi'an, China

**DOI:** 10.1002/alz70860_098744

**Published:** 2025-12-23

**Authors:** Simeng Cui, Qiumin Qu, Suhang Shang, Liangjun Dang, Jin Wang, Chen Chen, Ziyu Liu, Kang Huo, Wenhui Lu

**Affiliations:** ^1^ The First Affiliated Hospital of Xi'an Jiaotong University, Xi'an, Shaanxi, China; ^2^ The First Affiliated Hospital of Xi’an Jiaotong University, Xi'an, Shaanxi, China

## Abstract

**Background:**

To study the age‐dependent relationship between body mass index (BMI) and cognitive impairment in rural population aged 40 years and above.

**Method:**

From October 2014 to March 2015, people aged 40 years and above, lived in two natural villages in Huyi district of Xi'an, were selected as the research subjects. General demographic information, lifestyle, medical history, family history, physical examination, and biochemical examination were collected. Mini‐mental state examination (MMSE) assessed global cognitive function, with cognitive impairment defined as a score below the cutoff. The age‐dependent relationship between BMI and cognitive impairment was analyzed using stratified analysis, restricted cubic spline (RCS), and multivariate logistic regression.

**Result:**

A total of 1792 subjects were included in the analysis, of which 230 were diagnosed with cognitive impairment. There were 726 males (40.5%); the average age was 55.53±9.92 years, ranging from 40‐85 years, 1193 subjects aged 40‐59 years (66.6%), and 599 subjects aged ≥60 years (33.4%); the average BMI was 25.29±3.14 kg/m^2^. In the total population, logistic regression, adjusting for confounders, showed a significant correlation between BMI and cognitive impairment (*P*_overall_=0.023), with a nonlinear trend (*P*_nonlinear_=0.097); the specific relationship was that, for BMI <25 kg/m^2^, the odds ratio (OR) value increased as BMI decreased, while BMI ≥25 kg/m^2^ showed no significant change. The population was divided into two subgroups according to age (40‐59 years vs ≥60 years). Stratified analysis showed that in the ≥60 years old subgroup, the risk of cognitive impairment with BMI index was similar to that of the overall population (*P*_overall_=0.038, *P*_nonlinear_=0.097); but in the 40‐59 years old subgroup, BMI index was not significantly associated with cognitive impairment (*P*_overall_=0.722, *P*_nonlinear_=0.738).

**Conclusion:**

The relationship between BMI and cognitive impairment was affected by age. No significant association was found in the middle‐aged population of 40‐59 years old; while there may be a nonlinear association in the elderly population over 60 years old. Specifically, with BMI = 25 kg/m2 as the boundary, as BMI decreased, the risk of cognitive impairment gradually increased; and as BMI further increased, the risk of cognitive impairment did not change significantly even if it reached the obesity level.